# Valorization and Characterization of Agricultural and Forest Biomass Residues Through Colloidal Lignin Particle Production

**DOI:** 10.3390/polym18111352

**Published:** 2026-05-29

**Authors:** Julia Tomasich, Lukas Kaindl, Bastian Venclik, Sebastian Serna-Loaiza, Stefan Beisl, Michael Harasek, Richard Nadányi

**Affiliations:** 1 Circular Bioeconomy and Biorefineries Research Group, Institute of Chemical, Environmental and Bioscience Engineering, TU Wien, 1060 Vienna, Austria; lukas.kaindl@tuwien.ac.at (L.K.); richard.nadanyi@tuwien.ac.at (R.N.); 2 Research Unit of Technical Electrochemistry, Institute of Chemical Technologies and Analytics, TU Wien, 1060 Vienna, Austria; 3 Lignovations GmbH, 3400 Klosterneuburg, Austria

**Keywords:** renewable resources, biomass, lignin, colloids, biomaterials

## Abstract

The valorization of secondary biomass streams is an important step toward more resource-efficient biorefinery concepts and reduced dependence on fossil-based materials. In this study, agricultural and forest residues, namely Atlas cedar cones, mixed conifer cones, hazelnut shells, walnut shells, coffee silverskin, and cocoa shells, were investigated as feedstocks for producing colloidal lignin particles. Lignin-rich extracts were obtained by Organosolv pretreatment using 60 wt% aqueous ethanol, followed by particle formation through solvent shifting and purification by ultrafiltration. A particular novelty of this work is that highly different feedstocks were processed under identical Organosolv and solvent-shifting conditions, enabling a direct comparison of their suitability for colloidal lignin particle production within one consistent process route. The feedstocks differed markedly in extractive content and chemical profile, as shown by sequential Soxhlet extraction and qualitative GC-MS screening. Despite these differences in extract composition, solvent shifting yielded colloidal lignin particles with largely similar properties. Dynamic light scattering showed hydrodynamic diameters of 65–88 nm immediately after precipitation for all samples except cocoa shell, which formed strong agglomerates. The ultrafiltration step further introduced an industry-relevant downstream purification stage by removing most water-soluble low-molecular-weight compounds before product evaluation. After purification and redispersion, particle sizes ranged from 121 to 389 nm, indicating partial aggregation but overall successful recovery of stable colloidal dispersions. All purified particle suspensions exhibited comparable antioxidant activity in the FRAP (ferric reducing antioxidant power) assay, ranging from 12.3 to 18.4 mg lignin per mg ascorbic acid equivalents. These results demonstrate that even chemically diverse biomass side streams can be converted into purified colloidal lignin suspensions with similar colloidal behavior and functional performance. The findings highlight the potential of low-value agricultural and forest residues as promising raw materials for lignin-based antioxidant and material applications.

## 1. Introduction

A transition toward a bioeconomy is increasingly promoted as a strategy to reconcile economic development with climate-change mitigation and more sustainable patterns of production and consumption by replacing fossil resources with biomass in energy, food, and material systems. At the same time, this transition must be considered within existing biophysical limits, because further growth in biomass demand may intensify land-use competition, biodiversity loss, and greenhouse-gas emissions if it depends on additional primary biomass production rather than more efficient use of existing resources [1,2].

In this context, secondary biomass feedstocks are particularly relevant [3]. Agricultural and agro-industrial residues are generated as byproducts of existing production systems and can therefore add value without the same degree of additional pressure on land, water, and fertilizer inputs as dedicated biomass cultivation. Their valorization can also reduce waste generation and open new pathways for producing biofuels, biochemicals, and bio-based materials within integrated biorefinery concepts [4].

The feedstocks considered in this work fit well within this framework, namely Atlas cedar cones (*Cedrus atlantica*) and mixed conifer cones, hazelnut shells (*Corylus avellana*), walnut shells (*Juglans regia*), coffee silverskin (*Coffea arabica*), and cocoa bean shell (*Theobroma cacao*). These materials represent non-food lignocellulosic side streams from forestry and food-processing chains. Despite the heterogeneity of the materials, their common feature is a high lignin content [5,6,7,8]. Because they are generated at relatively concentrated points such as sawmills, nut-processing facilities, coffee roasters, in forests after harvesting and chocolate factories, they are attractive candidates for cascading valorization in biorefinery schemes. Previous studies have shown that such residues can serve as sources of fermentable carbohydrate fractions, phenolic-rich extractives, and solid fuels or carbonaceous products, supporting the production of bioactive compounds, platform chemicals, biofuels, and bio-based materials [7,9,10,11].

Organosolv pulping is a chemical fractionation approach that uses organic solvents, typically in combination with water and often an acid catalyst, to disrupt the recalcitrant lignocellulosic structure of biomass and selectively solubilize lignin and a substantial portion of hemicelluloses while preserving a cellulose-enriched solid fraction [12,13,14]. Common solvent systems include ethanol, methanol, acetone, acetic acid, and formic acid, with process conditions depending strongly on biomass type and targeted fractionation performance; for example, ethanol-based systems have been widely studied and also applied in technologies such as the Alcell process and the Lignol approach [12,14,15]. The key underlying mechanism involves cleavage of lignin ether linkages, especially α- and β-aryl ether bonds, through pathways such as nucleophilic substitution, quinone methide formation, and carbocation-mediated reactions, accompanied by hydrolysis of lignin–carbohydrate linkages and hemicellulosic glycosidic bonds; under more severe acidic conditions, released sugars may further degrade to furfural and 5-hydroxymethylfurfural [13]. In alcohol-based systems, the dissolved lignin fragments are subsequently recovered by dilution with water, precipitation, or solvent evaporation, yielding a sulfur-free, low-ash, relatively unmodified lignin of high purity that is generally more suitable for downstream valorization into chemicals, polymers, binders, or other materials than lignin isolated from conventional Kraft pulping [12,13,14,15]. At the same time, the process provides separated hemicellulosic streams and a cellulose-rich residue that can be further enzymatically hydrolyzed to fermentable sugars, making Organosolv fractionation particularly attractive from a biorefinery perspective [13,14].

Among the abovementioned lignocellulosic components, lignin attracts increased attention because of its complex phenolic structure that makes it suitable as fossil-based material replacement. Lignin is a three-dimensional, amorphous, highly heterogeneous aromatic biopolymer formed by oxidative coupling of the three primary monolignols, *p*-coumaryl alcohol, coniferyl alcohol, and sinapyl alcohol, which give rise to the H, G, and S structural units, respectively [16,17,18]. Its macromolecular architecture is dominated by ether linkages—especially the β-O-4 bond—together with condensed C-C linkages such as β-5, β-β, β-1, and 5-5, and the relative abundance of these bonds varies with biomass origin; in general, higher G content is associated with a more condensed structure, whereas S-rich lignin tends to contain a larger fraction of more labile ether linkages [12,16,17,18]. From a functional-group perspective, lignin contains phenolic hydroxyl, aliphatic hydroxyl, methoxy, carbonyl, and carboxyl moieties, which govern its polarity, reactivity, intermolecular interactions, and suitability for chemical modification [16,17,18]. This structural complexity underlies a set of highly attractive properties, including aromaticity, relatively high carbon content, antioxidant and antimicrobial activity, UV absorption, thermoplastic/thermosetting behavior depending on temperature and crosslink density, and glass-transition temperatures commonly reported in roughly the 95–162 °C range for technical lignins, with literature often placing lignin-based material behavior broadly within about 90–180 °C depending on origin and processing [16,18,19]. A further practical advantage is that different technical lignins offer distinct application windows: kraft lignin is the most abundant commercial source; lignosulfonates are water-soluble and surfactant-like; Organosolv lignin is sulfur-free, relatively pure, and chemically reactive; and enzymatically isolated lignins are often closer to native structure and therefore valuable when structure preservation is important [16,17,18,19]. Taken together, lignin is especially compelling because it is one of the largest renewable sources of aromatic carbon, is generated in very large quantities yet remains strongly underutilized, can replace selected fossil-derived phenolic and aromatic feedstocks, and fits naturally into circular biorefinery concepts in which a low-value byproduct is upgraded into chemicals, functional materials, and bio-based additives [12,16,17,18,19].

Colloidal lignin particles (CLPs) are nanoscale-to-submicron particulate forms of lignin obtained by inducing controlled self-assembly of dissolved lignin molecules, typically through antisolvent precipitation, and they are attractive because they render lignin water-dispersible without requiring extensive chemical derivatization. Their formation is generally attributed to cooperative non-covalent interactions, mainly π-π stacking between aromatic moieties, hydrophobic association, and hydrogen bonding, which drive lignin molecules to reorganize into colloidal structures. Among the available preparation routes, solvent exchange/antisolvent precipitation is the most widely used, usually by dissolving lignin in an aqueous organic solvent and then adding water to reduce solubility and trigger particle nucleation and growth; process variables such as initial lignin concentration, final solvent content, antisolvent pH, and addition rate strongly affect particle size, zeta potential, hydrophobicity, and stability [20,21]. Beyond classical nanoprecipitation, the literature also reports related routes such as dialysis, acid precipitation, aerosol/mechanical routes, and self-assembly-assisted capsule formation or crosslinking/stabilization strategies, depending on the intended end use [18,20,21,22]. CLPs have been prepared from a broad range of lignins, including softwood kraft lignin, Organosolv lignin, eucalyptus lignin isolated by enzyme/alkali/Organosolv treatments, lignin from enzymatic hydrolysis residues such as corn stover, and lignin-rich fractions from agro-industrial side streams such as cocoa shells [20,21,22]. Their major applications already span Pickering emulsions, cosmetic and skincare formulations, encapsulation and drug-delivery systems, antimicrobial platforms, durable coatings, UV-protective materials, adsorption/water-treatment systems, composite reinforcement, adsorption technologies, and protective coatings [18,20,21,22,23].

In the attached studies, CLPs stabilized fully emulsified oil-in-water Pickering systems with month-long storage stability for cosmetic-type formulations [20], and CLP-epoxy systems produced thin, durable, abrasion-, heat-, water-, stain-, and sunlight-resistant bio-based coatings on wood [22]. From a biorefinery viewpoint, this is particularly relevant for Organosolv lignins because their higher purity, lower sulfur content, and better-defined chemistry make them promising precursors for downstream CLP manufacture [18,19,20,21]. While the initial extract composition varies significantly with biomass origin, the precipitation process yields particles that exhibit comparable colloidal behavior and functional performance.

## 2. Methods

### 2.1. Feedstock Characterization

The agricultural and forest residues were ground using either a cutting mill or a jaw crusher, depending on their hardness. Softer materials, including coffee silverskins, Atlas cedar cones, mixed conifer cones, and cocoa shells, were milled by a cutting mill (Hosakawa Alpine, Augsburg, Germany) using a 2 mm sieve insert to obtain uniform particle sizes. Walnut and hazelnut shells were processed with a jaw crusher to achieve comparable particle sizes of approximately 1–3 mm. Moisture content of the raw materials was determined using a thermogravimetric moisture analyzer (Sartorius MA150, Satarius AG, Göttingen, Germany).

#### Soxhlet Extraction

Solid–liquid extraction was performed in duplicates based on Determination of Extractives in Biomass: Laboratory Analytical Procedure NREL/TP-510-42619 [24] following the principles of Soxhlet extraction method [25]. Briefly, 10 g of milled material were placed in a cellulose thimble. Sequential extractions were conducted with water (300 g) and absolute ethanol (192 g) to obtain the respective soluble fractions. Each extraction was carried out for 24 h under total reflux. The extracts were cooled to room temperature, after which an aliquot was collected and stored at −20 °C for subsequent gas chromatography–mass spectrometry (GC–MS) analysis. The remaining solutions were evaporated to dryness under reduced pressure at 40 °C for quantification.

For GC-MS prior to injection, the samples were derivatized with a mixture of 80 µL BSTFA, 20 µL TMCS, and 20 µL pyridine per sample. The reaction was carried out in a drying oven at 70–75 °C for 45 min.

Gas chromatography–mass spectrometry (GC–MS) analysis was performed using an Agilent GC coupled to an electron-impact ionization mass spectrometer operating in full-scan mode. Samples (1 µL) were injected in split mode (5:1) through a heated inlet at 280 °C. Separation was achieved on an HP-5MS capillary column (30 m × 0.25 mm × 0.25 µm, Agilent) under constant helium flow (1.34 mL min^−1^). The oven program started at 100 °C (1 min hold), followed by heating at 10 °C min^−1^ to 325 °C, with a final 15 min isothermal hold (total run time: 38.5 min). The MS transfer line was maintained at 280 °C.

Mass spectrometry data were acquired in scan mode (m/z 35–750) with a solvent delay of 6.5 min. The ion source and quadrupole temperatures were set to 230 °C and 150 °C, respectively. Data acquisition was performed at a rate of 20 Hz, and compound identification was based on comparison with the NIST mass spectral libraries. The accordance of spectral identity was >85% for a substance to be taken into account.

### 2.2. Process

#### 2.2.1. Lignin Extraction via Organosolv Process

Organosolv extraction was performed to isolate lignin from the different untreated lignocellulosic residues. The process was carried out in a 1 L stirred autoclave (Zirbus, HAD 9/16, Bad Grund, Germany) using a 60 wt% aqueous ethanol solution as the solvent, accounting for the feedstock’s moisture content. The biomass loading, based on dry matter, was 8.3 wt%. The reactor was heated to 180 °C and maintained at this temperature for 60 min under continuous stirring.

After completion, the reactor was cooled to room temperature using an external water-cooling system. The resulting suspension was separated into solid and liquid fractions using a hydraulic press (Hapa, HPH 2.5, Achern, Germany) operated at 200 bar. The liquid extract was subsequently centrifuged (Thermo Scientific, Sorvall RC 6+, Waltham, MA, USA) at 24,104× *g* for 20 min to remove residual solids.

Two extraction batches were performed, and the particle-free extracts were combined after centrifugation to minimize sample variability. The combined extracts were stored at 5 °C until further compositional analyses and lignin nanoparticle precipitation experiments were performed.

#### 2.2.2. Precipitation of Colloidal Lignin Particles

The Organosolv extracts were diluted to a uniform concentration to ensure comparability among samples. Dilution was performed using a 60 wt% aqueous ethanol solution prepared from absolute ethanol and deionized water to a dry matter content of 0.83 wt%. Colloidal lignin particles (CLPs) were produced by a solvent-shifting method in which deionized water (18 MΩ/cm^2^, Sartorius arium pro system) was added to the diluted extracts at a fixed volume ratio of 5:1 (water to extract). The precipitation setup is described in Beisl et al. [26]. Briefly, water and the extract were pumped using a syringe pump that used five syringes for water and one for the extract. These fluids were directed into a T-shaped flow reactor, where precipitation occurred at 25 °C. Three precipitation runs were conducted for each extract to ensure reproducibility. Following precipitation, the suspensions were collected and stored for further purification, yield determination, and compositional analysis.

#### 2.2.3. Purification and Concentration of CLP Suspensions

The colloidal lignin particle (CLP) suspensions were purified by ultrafiltration using a polyethersulfone (PES) membrane with a 30 kDa molecular weight cutoff (supplied bei Nadir ^®^, Wiesbaden, Germany). Purification is understood here as the removal of carbohydrates from the CLP dispersion originally dissolved in the Organosolv extract. The process was performed under compressed air pressure (6–8 bar) to separate the permeate from the concentrated lignin suspension (retentate). After purification, both fractions were collected for further analysis.

### 2.3. Characterization Methods

#### 2.3.1. Organosolv Extracts

The Organosolv extracts were analyzed for carbohydrates and lignin. The acid-insoluble and acid-soluble lignin fractions were determined according to the NREL LAP “Determination of Structural Carbohydrates and Lignin in Biomass” [27]. Carbohydrate quantification followed the NREL laboratory analytical procedure for “Determination of Sugars, Byproducts, and Degradation Products in Liquid Fraction Process Samples”, with minor modifications—specifically, samples were not neutralized after hydrolysis [28]. Individual sugars (arabinose, glucose, mannose, xylose, and galactose) were quantified using high-performance anion-exchange chromatography with a pulsed amperometric detector (HPAEC-PAD; ICS5000, Thermo Scientific, USA) using deionized water as the eluent.

#### 2.3.2. Colloidal Lignin Particles

The hydrodynamic diameter and particle size distribution of the non-purified suspension of colloidal lignin particles (CLPs) were determined by dynamic light scattering (DLS) using a Litesizer 500 (Anton Paar GmbH, Graz, Austria). Measurements were performed on aqueous suspensions with the refractive index set to 1.53 and the imaginary refractive index to 0.1. Reported values represent the mean ± standard error of three independent measurements, based on intensity-weighted distributions.

#### 2.3.3. Purified Colloidal Lignin Particles

The dry matter content of the initial suspension, permeate, and retentate was determined gravimetrically after drying at 105 °C to constant weight. Electrical conductivity of the permeates was measured to assess washing efficiency. The purified and concentrated CLP suspensions were then homogenized under high shear force using Ultra Turrax T25 equipped with the dispersing tool S 25 KD—25 G (IKA-Werke GmbH&Co.KG, Staufen, Germany) and analyzed by dynamic light scattering (DLS) for particle size determination.

Further, the colloidal lignin particles were analyzed for carbohydrates and lignin using the same NREL analytical procedure described in point 2.4. These data were used to determine the precipitation yield and to track the distribution of different molecular components throughout the precipitation process.

#### 2.3.4. Antioxidative Behavior of Colloidal Lignin Particles by FRAP Method

The antioxidant properties of the colloidal lignin particle suspensions were evaluated using the FRAP (Ferric Reducing Antioxidant Power) assay, following a modified procedure based on Benzie and Strain [29]. A calibration curve was prepared using five ascorbic acid standards (0.01–0.30 g/L). The FRAP reagent was freshly prepared by combining 300 mM acetate buffer, 10 mM TPTZ in 40 mM HCl, and 20 mM FeCl_3_·6H_2_O in a 10:1:1 volumetric ratio. For each measurement, 3 mL of FRAP reagent was mixed with 100 µL of sample or standard in disposable cuvettes and incubated for 6 min, after which the absorbance at 593 nm was recorded. All measurements were performed in triplicate, and mean values were used for data evaluation. Results are given as ascorbic acid (ASC) equivalents.

## 3. Results

### 3.1. Characterization of Lignocellulosic Feedstock

To assess the amount of extractable compounds present in the studied biomass residues, the liquid fractions obtained after sequential Soxhlet extraction were evaporated to dryness, and the dry mass of each extract was determined. Using the dry matter content of the corresponding raw materials (Supplementary Material, Table S1) as the reference, the proportions of water-soluble and ethanol-soluble extractives were calculated and are summarized in Table 1. Considerable differences were observed among the feedstocks. Atlas cedar cones showed a moderate total extractive yield of 13.64 wt%, with substantially higher recovery in water (9.42 wt%) than in ethanol (4.22 wt%). Mixed conifer cones exhibited a lower total extractive content (7.51 wt%), with similarly low yields in both solvents. This is generally consistent with literature reports for conifer cone materials, although published extractive contents vary considerably among species and anatomical fractions [30,31]. Hazelnut shells and walnut shells yielded comparatively low amounts of extractives. Hazelnut shells contained 4.38 wt% total extractives, while walnut shells yielded 5.11 wt% water-soluble extractives and no gravimetrically detectable ethanol-soluble fraction. Although no ethanol-soluble extractives were measured by weight in walnut shells, GC–MS analysis showed a few ethanol-soluble compounds, implying the extract yield was very low but still contained some chemical compounds. Such low extractive contents are in line with the dense and highly lignified nature typically reported for nutshell-derived materials. At the same time, extraction efficiency may also be influenced by methodological parameters such as particle size, which should be considered when comparing values across studies. In contrast, coffee silverskins and cocoa shells exhibited markedly higher extractive contents, reaching 27.35 and 51.28 wt%, respectively. These results indicate that both residues contain a large fraction of solvent-accessible low-molecular-weight compounds. Reported extraction yields for these feedstocks vary widely in the literature, which is likely related to differences in raw material origin, pretreatment, solvent system, extraction procedure, and the way results are expressed [32,33,34].

Qualitative GC–MS screening of the Soxhlet extracts further revealed pronounced feedstock-dependent differences in chemical composition (Tables S2 and S3). In general, the aqueous extracts were characterized mainly by polar low-molecular-weight constituents, including organic acids, sugar acids, sugars and sugar alcohols, amino acids and their derivatives, and selected polar phenolics. In contrast, the ethanol extracts contained a less polar set of compounds, especially fatty acids, resin acids and other diterpenoid compounds, sterols, and selected phenolic constituents. This overall trend is consistent with the expected solvent selectivity of the sequential extraction procedure.

The cone-derived samples, particularly Atlas cedar cones and mixed conifer cones, showed a characteristic conifer-type extractive profile. Their ethanol extracts were distinguished by the presence of resin acids and related diterpenoids, including abietic acid, dehydroabietic acid, isopimaric acid, neoabietic acid, palustric acid, and pimaric acid, together with oxidized derivatives in some cases. These compounds are typical for conifer-derived biomass and point to the presence of a lipophilic resin-rich fraction with potential relevance for further valorization. In comparison, the water extracts of the cone materials contained mainly organic acids, sugar acids, and sugars/sugar alcohols, with only limited detection of resin-acid-related compounds. Atlas cedar cones additionally showed quinic acid, citric acid, shikimic acid, and dehydroabietic-type compounds in the aqueous extract, indicating a broader range of water-extractable metabolites than the mixed conifer cone sample.

The nutshell samples exhibited lower extractive yields and a narrower qualitative GC–MS profile. In hazelnut shells, the aqueous extract contained mainly sugars, sugar-derived acids, and phenolic acids such as gallic, protocatechuic, and vanillic acid, whereas the ethanol extract showed fatty acids together with trans-coniferyl alcohol. Walnut shells displayed a similarly limited profile, with vanillic acid detected in the water extract and trans-coniferyl alcohol together with palmitic acid in the ethanol extract.

Although no ethanol-soluble extractives were gravimetrically detected for walnut shells, the GC–MS screening still indicated the presence of a limited number of ethanol-soluble compounds, suggesting that the mass of the extract was very low but not chemically devoid of identifiable constituents. Overall, the nut shells appear to contain a relatively small extractable fraction composed mainly of simple polar metabolites and selected phenolic or lignin-related aromatic compounds.

Coffee silverskins and cocoa shells were clearly the most extractive-rich and chemically diverse materials in the present set. Their aqueous extracts contained a broad range of compounds, including caffeine, amino acids and related metabolites, sugar acids, sugars, and sugar alcohols. In cocoa shells, this polar fraction was especially diverse, with multiple amino acids, organic acids, catechin, caffeine, and several sugar-derived compounds detected. The ethanol extracts of both materials were characterized by the presence of fatty acids such as palmitic, stearic, oleic, and linoleic acid, while coffee silverskin additionally contained very-long-chain fatty acids and β-sitosterol. These findings indicate that both residues contain not only a substantial polar extractive fraction but also a chemically relevant lipophilic fraction. From a valorization perspective, the occurrence of caffeine and catechin in cocoa shells, and caffeine, sterols, and long-chain fatty acids in coffee silverskin, is particularly notable because these compounds are commonly associated with bioactive or functional applications.

Taken together, the results demonstrate that the studied residues cannot be treated as a chemically uniform group of biomass byproducts. Instead, they represent distinct extractive reservoirs that differ markedly in both total extractive yield and compound class distribution. The cone-derived materials were characterized primarily by resin acids and diterpenoid-type extractives, the nut shells by comparatively low extractive yields and a limited set of phenolic and fatty-acid-related compounds, and coffee silverskins and cocoa shells by high extractive contents and the broadest chemical diversity. At the same time, it should be emphasized that the present GC–MS data provide only a qualitative compositional fingerprint. Therefore, the detected compounds should be interpreted as solvent-extractable constituents identified under the applied derivatization and analytical conditions, rather than as a measure of their absolute abundance in the raw materials. Nevertheless, these findings are relevant for downstream processing because extractives may influence the efficiency of subsequent fractionation steps as well as the purity and properties of isolated lignin fractions. On this basis, the following section focuses on lignin extraction and its further valorization.

### 3.2. Organosolv Extract Composition

The Organosolv pretreatment employs aqueous organic solvents at elevated temperatures to disrupt the lignin–carbohydrate complex and solubilize lignin [35,36]. In this study, a 60 wt% aqueous ethanol mixture was used at 180 °C, yielding lignin-rich extracts from all tested feedstocks. The concentrations of all compounds extracted by the Organosolv process (g/L) for the different untreated biomass feedstocks are presented in Table 2. The total extract concentrations ranged from approximately 11 to 26 g/L, reflecting substantial differences between the biomass types. Similar to the Soxhlet extraction results, the nut shells exhibited the lowest extraction efficiencies, followed by moderate yields for the conifer cones. In contrast, the coffee silverskin and cocoa shell extracts showed nearly twice the overall solute concentration compared to the nut-based feedstocks.

These trends are consistent with literature reports indicating that the efficiency of Organosolv extraction is strongly influenced by the structural characteristics of the feedstock, including cell wall architecture and tissue composition, as well as by the particle size of the milled material [33]. The lignin concentrations in the Organosolv extracts were within a comparable range across all feedstocks, approximately 8.5–12 g/L (Table 2). This limited variation is likely attributable to the underlying anatomical and physicochemical properties of the materials. Factors such as porosity, tissue density, and extractive content influence solvent accessibility and penetration, thereby affecting the extent to which lignin can be solubilized and recovered during the Organosolv process [37].

To further discuss the composition of the extracts, units for the composition of the extracts are converted from concentration (g/L) to weight percent (wt%) to enable comparison and evaluation of the lignin extraction yield from the feedstocks.

**Figure 1:**
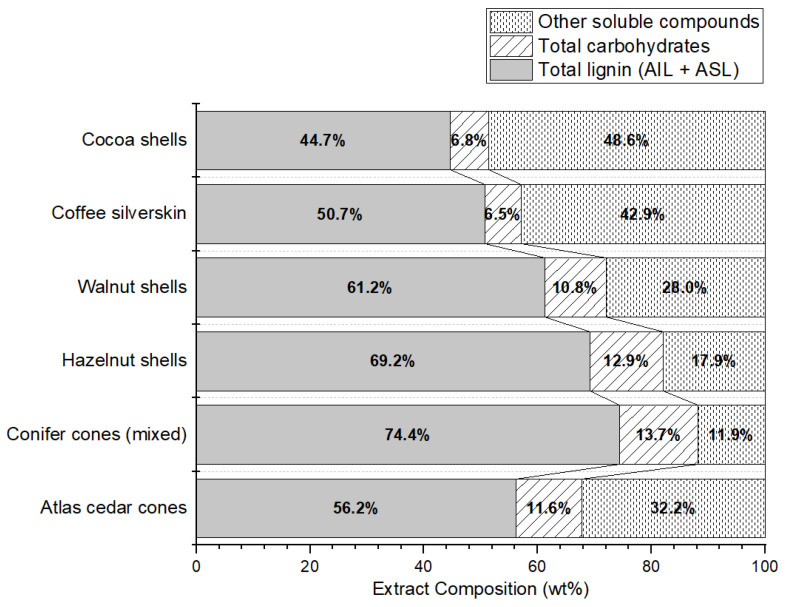
Extract composition in wt% of the Organosolv extracts (dried) obtained from agricultural and forest feedstocks.

The composition of the extracts, plotted in Figure 1, shows significant differences: the obtained lignin concentrations were very high in the mixed conifer cones and hazelnut shells, followed by walnut shells and atlas cedar cones. The lowest lignin percentages were reached for coffee silverskins and cocoa shells. The “other” label refers to unidentified compounds resulting from the protocols used to analyze the composition, in accordance with the lab procedures described in the methods section.

When these data are linked to the Soxhlet extraction outcomes, it makes sense, as the coffee silverskins and cocoa shells, as well as the atlas cedar cones, show high extraction concentrations with both solvents, and a wide variety of extract types is confirmed via GC/MS.

Table 3 summarizes the lignin extraction yields obtained from the agricultural and forest residues, together with literature-reported lignin contents for these biomass types. The lignin extraction yield, based on dry feedstock, ranges from 9 to 15 wt%, indicating that, on a mass basis, the Organosolv process recovered broadly comparable amounts of lignin-containing material from all investigated feedstocks. In contrast, when the yields are normalized to the estimated lignin content of the respective raw material, the calculated recovery spans a substantially wider range, from 21 to 40% for walnut shells and 28–50% for mixed conifer cones, up to 53% for coffee silverskins and cocoa shells, and 37% for hazelnut shells.

This difference between yield per dry feedstock and yield per lignin content highlights that extraction efficiency depends not only on process conditions, but also on feedstock-intrinsic factors such as lignin accessibility, tissue density, and the presence of extractives and other soluble components that influence solvent penetration and mass transfer. Consequently, similar extraction yields on a dry matter basis can correspond to markedly different degrees of lignin recovery from the native biomass matrix. For atlas cedar cones, a comparable normalization could not be calculated due to the lack of a reliable lignin-content value in the available literature dataset.

### 3.3. Colloidal Lignin Particles Produced from Organosolv Extracts

#### 3.3.1. Colloidal Lignin Particles After Precipitation

**Figure 2:**
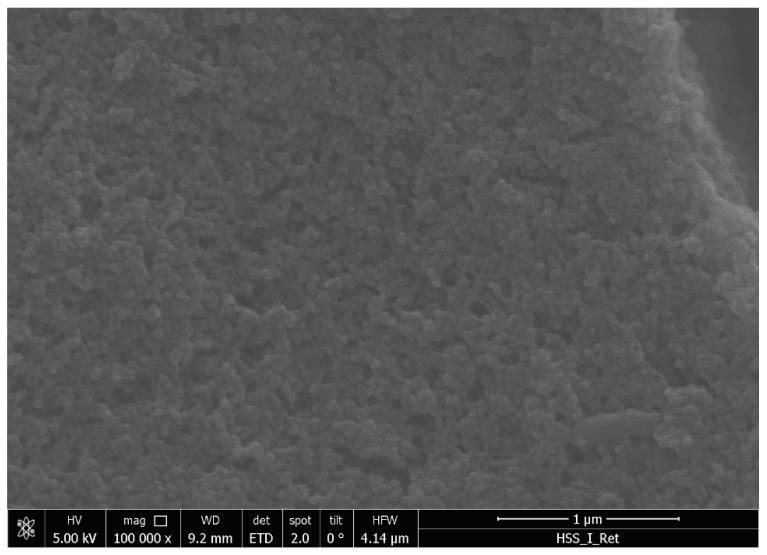
SEM Image of colloidal lignin particles produced from Organosolv extractives of Hazelnut shells; magnification: 100,000×.

In the present work, CLP-formation was achieved via solvent-shifting, driven by lignin supersaturation in the solvent system, leading to the precipitation of nanoscale particles with a hydrophobic core and a hydrophilic shell [41]. DLS was used to determine the hydrodynamic diameter and polydispersity of the CLP suspension formed after precipitation. Table 4 summarizes the results of the DLS measurements. Hydrodynamic diameters ranged from 65 to 88 nm and polydispersity from 18.0 to 22.7%, whereas CLPs originating from cocoa shell extract showed substantial differences, with hydrodynamic diameters of approximately 4750 nm and a polydispersity of 34.8%. Therefore, the hydrodynamic diameters of CLP suspensions produced lie, with the exception of the cocoa shell CLPs, within the definition of nanoparticles, ranging from 1 to 100 nm [42]. The differences observed for the cocoa shell CLPs do not arise from actual differences in particle size, but rather from the tendency of nano-scaled CLPs to form agglomerates caused by high surface energy, large numbers of hydrogen bonds, and Van der Waals forces [43]. Scanning electron microscopy after purification revealed nanoscale primary particles and aggregates formed from them (Figure 2 and Figure S1), independent of the extract’s initial raw material, thus confirming agglomeration.

#### 3.3.2. Process Yield

**Figure 3:**
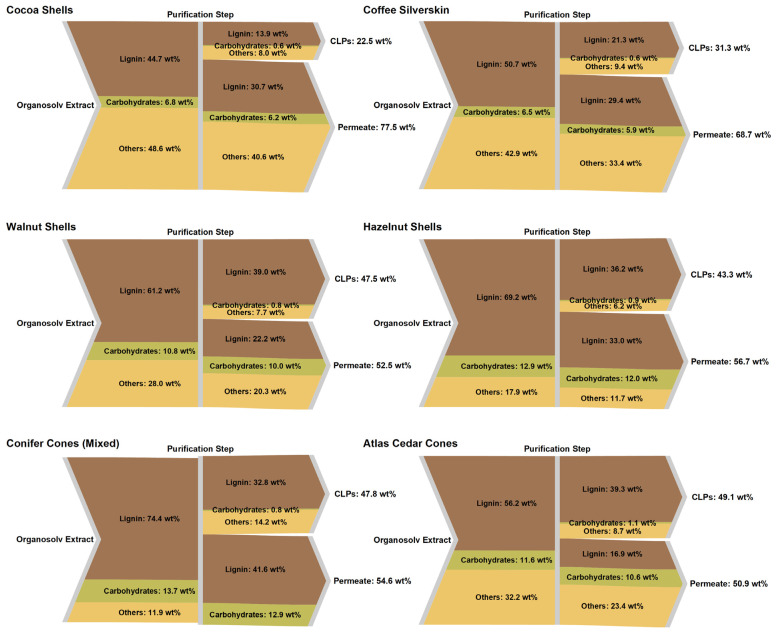
Sankey diagrams of CLP production out of Organosolv extracts based on dried extract.

Precipitated CLP suspensions were purified via ultrafiltration to remove dissolved components which are not part of CLPs, especially carbohydrates. Material yields of the purification steps varied across the Organosolv extracts (Figure 3), ranging from 22.5 wt% to 49.1 wt%. The lowest yields in this step were observed for cocoa shells and coffee silverskins, at 31.3 wt% and 22.5 wt%, respectively, whereas yields for the other extracts were significantly higher. Therefore, higher initial extraction yields for coffee silverskins and cocoa shells do not necessarily translate into higher CLP yields. Figure 3 depicts the purification losses, illustrating significant losses of extractives from cocoa shells and coffee silverskins that are neither carbohydrates nor lignin. The overall amount of CLPs yielded in this process ranges from approximately 5.4 wt% (hazelnut shells) to 8.3 wt% (atlas cedar cones) based on the initial feedstock (oven dry basis).

**Figure 4:**
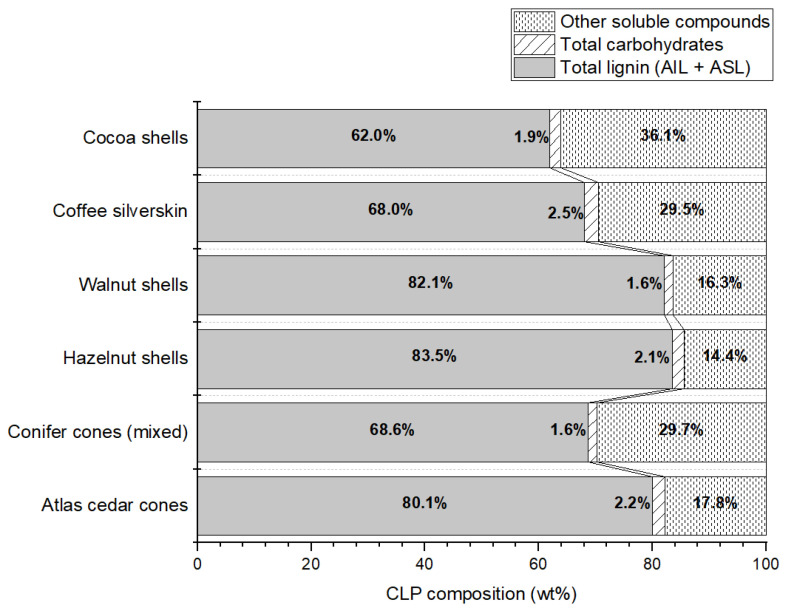
Composition of the CLPs (dried) obtained from agricultural and forest feedstocks.

Thus, relatively large material losses must be accepted in the current process design, which, however, effectively removes carbohydrates and, in most cases, increases the overall lignin content of the suspensions (Figure 4). Nonetheless, it is worth noting that solubilized lignin is also lost during purification, indicating that lignin fractions smaller than the ultrafiltration membrane’s cut-off are removed in the process. Conductivity measurements (Table S8) of the removed, particle-free permeates after each purification step revealed a relationship between the “others” content in the extract and the permeate’s conductivity. Conifer cones (mixed) and hazelnut shells with the lowest content of other extracts showed the lowest conductivity, whereas the opposite was observed for cocoa shells and coffee silverskins samples, which contained the highest amount of other extractives.

#### 3.3.3. Particle Size After Redispersion

After purification, CLPs are present as CLP aggregates. Thus, homogenization was applied to disintegrate these aggregates again. Once more, DLS was used to determine the hydrodynamic diameter and polydispersity of the CLP dispersions, confirming successful redispersion in aqueous media through mechanical treatment (Table 5). Nonetheless, compared to measurements following the precipitation step, hydrodynamic diameters ranged from 121 to 389 nm and were therefore larger. Furthermore, overall polydispersity increased compared to the non-purified CLP suspensions, ranging from 21.7% to 31.3%. This indicates that, after redispersion, the CLPs remain as agglomerates, albeit relatively small ones. However, agglomerates detected for the CLPs derived from cocoa shell extract after precipitation were successfully reduced to smaller sizes through high shear force homogenization.

#### 3.3.4. Antioxidant Activity of Purified Colloidal Lignin Particles

Lignin’s antioxidant activity is primarily attributed to the availability of free phenolic hydroxyl groups, which are further promoted by other functional groups, such as *o*-methoxy groups or conjugated double bonds [44]. Due to high surface-to-volume ratios and a high degree of phenolic hydroxyl groups concentrated towards the particle surface, CLPs show superior antioxidant properties, even outperforming solubilized lignin [45]. This makes CLPs a promising resource for the application as a sustainable antioxidant. Here, the antioxidant activity of CLPs was determined following the FRAP assay using solid colloidal lignin particles.

Results are summarized in Table 6, expressed as equivalents of ascorbic acid (ASC). Overall, similar antioxidant activities were observed across all CLP types, ranging from 12.3 to 18.4 mg CLP/mg ASC equivalents. Adamcyk et al. [36], who followed the same procedure characterizing the antioxidant activity of Organosolv lignin-based CLPs (wheat straw, beech wood, spruce wood), observed significantly higher ASC equivalents in the range of 19.1 to 50.4 mg Lignin/mg ASC. The comparatively low ASC equivalents and correspondingly high antioxidant activity of the particles from the selected raw materials in the present study raise the possibility of using those CLPs as antioxidants, which cannot be applied in solubilized form but require application in the solid state, such as in dermocosmetics [45].

Although the literature confirms an influence of the CLPs’ particle size and the corresponding specific surface area on the antioxidant activity [46], no relation between those parameters was found in this work. This could be attributed to the presence of lignin particles in the form of agglomerates, represented in the hydrodynamic diameter, consisting of several smaller primary particles (Figure S1), and to the fluctuating composition of the CLPs due to different raw material utilization (Figure 4).

## 4. Conclusions

Lignin-rich extracts were successfully produced using the Organosolv process. The effects of particle size and tissue properties of agricultural and forest residues are logical and significant. These extracts were characterized and found to contain fluctuating fractions of carbohydrates, lignins, and other components. This variation is due to differences in feedstock composition and the ability to access lignin or carbohydrates during pretreatment, influenced by particle size and tissue properties. The “other” components, such as extractives, were identified through Soxhlet extraction followed by GC/MS analysis. Organic acids and sugar monomers might solubilize and become part of the extract after pretreatment. A particular novelty of this work is that highly different feedstocks were subjected to the same Organosolv conditions and the same solvent-shifting process, allowing a direct comparison of their behavior within one consistent processing route. Despite the clear differences in extract composition, the downstream precipitation process yielded colloidal lignin particles with remarkably similar characteristics, highlighting the robustness of the approach across chemically diverse raw materials. In addition, membrane purification was included as an industry-relevant downstream step to remove most water-soluble low-molecular-weight compounds from the suspensions before further evaluation. After purification and redispersion, the particles showed comparable hydrodynamic diameters, while SEM confirmed a similar morphology across feedstocks. The purified suspensions also exhibited comparable antioxidant behavior, demonstrating that functional CLP dispersions can be obtained even from strongly differing biomass sources. Beyond their antioxidant properties, the produced CLPs may also be promising for future application in functional materials such as antibacterial coatings, protective surface treatments, or bio-based composite systems. Their comparable particle properties across different feedstocks support their potential use as versatile lignin-based building blocks for a broader range of material applications. Overall, these results show that diverse agricultural and forest residues can serve as suitable feedstocks for the production of comparable colloidal lignin particles. Thus, the study demonstrates not only the versatility of the process route but also its relevance for future lignin valorization concepts that aim to integrate variable side streams into robust and application-oriented material production.

## Figures and Tables

**Table 1:** Quantification of extractable compounds of lignocellulosic residues after sequential Soxhlet extraction by water and ethanol (abs).

Raw Material	Water-Soluble Extractives (wt%)	Ethanol-Soluble Extractives (wt%)	Soluble Extractives (Total) (wt%)
Atlas cedar cones	9.42	4.22	13.64
Conifer cones (mixed)	3.60	3.90	7.51
Hazelnut shells	2.65	1.73	4.38
Walnut shells	5.11	n.d.	5.11
Coffee silverskins	17.82	9.53	27.35
Cocoa shells	32.90	18.38	51.28

**Table 2:** Concentration in g/L of the Organosolv extracts obtained from agricultural and forest feedstocks.

	Lignin (g/L)	Carbohydrates (g/L)	Other Soluble Compounds (g/L)	Total Soluble Compounds (g/L)
Atlas cedar cones	8.58	1.77	4.91	15.27
Conifer cones (mixed)	10.76	2.11	1.59	14.47
Hazelnut shells	7.85	1.46	2.03	11.33
Walnut shells	8.96	1.57	4.10	14.62
Coffee silverskins	11.96	1.52	10.12	23.60
Cocoa shells	11.49	1.74	12.49	25.71

**Table 3:** Lignin yield from Organosolv process based on the dry feedstock and literature-reported lignin content [31,32,34,38,39,40].

	Extracted Lignin (wt% of Dry Feedstock)	Lignin Content (wt% According to Literature)	Calculated Lignin Extraction Yield (wt% of Lignin Content **)
Atlas cedar cones	10.72	*	*
Conifer cones (mixed)	13.51	27–48	28–50
Hazelnut shells	9.42	25	37
Walnut shells	10.96	27.4–52.3	21–40
Coffee silverskins	15.19	28.6	53
Cocoa shells	14.48	26–27.5	53

* no values found in the literature. ** according to the literature.

**Table 4:** Hydrodynamic diameter and polydispersity results following DLS measurements after CLP precipitation (3 measurements).

	Hydrodynamic Diameter (nm)	Polydispersity (%)
Atlas cedar cones	70 ± 4	19.6 ± 6.4
Conifer cones (mixed)	68 ± 4	18.0 ± 3.2
Hazelnut shells	66 ± 6	22.7 ± 4.3
Walnut shells	65 ± 1	18.1 ± 0.8
Coffee silverskins	88 ± 26	22.3 ± 1.3
Cocoa shells	4748 ± 1047	34.8 ± 8.0

**Table 5:** Hydrodynamic diameter and polydispersity determined via DLS after purification (3 measurements).

	Hydrodynamic Diameter (nm)	Polydispersity (%)
Atlas cedar cones	304 ± 205	24.8 ±6.1
Conifer cones (mixed)	121 ± 7	21.7 ± 3.6
Hazelnut shells	168 ± 32	26.6 ± 4.0
Walnut shells	157 ± 11	24.6 ± 2.2
Coffee silverskins	389 ± 102	31.3 ± 5.0
Cocoa shells	184 ± 98	27.7 ± 10.2

**Table 6:** Antioxidant activity of CLPs determined via the FRAP assay.

	FRAP ASC Equivalent (mg Lignin/mg ASC)
Atlas cedar cones	14.2 ± 3.2
Conifer cones (mixed)	12.3 ± 1.4
Hazelnut shells	16.2 ± 1.5
Walnut shells	14.9 ± 1.4
Coffee silverskins	18.4 ± 2.0
Cocoa shells	13.7 ± 1.0

## Data Availability

The original contributions presented in this study are included in the article and Supplementary Materials. Further inquiries can be directed to the corresponding authors.

## Supplementary Materials

The following supporting information can be downloaded at: https://www.mdpi.com/article/10.3390/polym18111352/s1. Table S1: Dry Matter of agricultural residue feed stocks; Table S2: Chemical compounds found in the aqueous extracts (Soxhlet) and organized by chemical group via GC/MS; Table S3: Chemical compounds found in the ethanol extracts (Soxhlet) and organized by chemical group; Table S4: Density of Organosolv extracts determined by Density Meter DE45 Delta Range™ (Mettler Toledo); Table S5: Concentration in g/L of the Organosolv extracts obtained from agricultural and forest feedstocks; Table S6: Lignin composition (wt%) of the Organosolv extracts obtained from agricultural and forest feedstocks; Table S7: Carbohydrate composition (wt%) of the Organosolv extracts obtained from agricultural and forest feedstocks; Figure S1: SEM pictures of (a) Atlas cedar cones, (b) Conifer cones (mixed), (c) Hazelnut shells, (d) Walnut shells, (e) Coffee silverskins and (f) Cocoa shells; Table S8: Conductivity Values of the permeates after ultrafiltration; Table S9: Overall CLP production yield.
