# Supplementary material for: Valorization and Characterization of Agricultural and Forest Biomass Residues Through Colloidal Lignin Particle Production
Source: Polymers (Basel). 2026 May 29;18(11):1352. doi: 10.3390/polym18111352 (PMC13259322; doi:10.3390/polym18111352)
Supplement: Supplementary file 1 [file polymers-18-01352-s001.zip › polymers-4322630-supplementary.pdf]

Table S1: Dry Matter of agricultural residue feed stocks

	Dry Matter (%)
Atlas cedar cones	93.35
Conifer cones (mixed)	93.26
Hazelnut shells	91.03
Walnut shells	94.97
Coffee silverskin	93.94
Cocoa shells	95.98

Table S2: Chemical compounds found in the aqueous extracts (Soxhlet) and organized by chemical group via GC/MS

Group	Compound	Atlas cedar cones	Mixed Conifer cones	Hazel-nut shells	Walnut shells	Coffee silver-skin	Cocoa shells
Alkaloids	Caffeine					X	X
Amino acids and derivatives	4-Aminobutanoic acid	X					
	4-Aminobutyric acid						X
	5-Oxoprolin					X	
	Aspartic acid					X	X
	Homoserine						X
	L-5-Oxoproline		X				
	L-Glutamic acid						X
	L-Lysine						X
	L-Pyroglutamic acid	X					X
	L-Threonine						X
	Phenylalanine						X
	Pipecolic acid						X
	Serine						X
	Threonine					X	
Aromatic compounds	(1H-indol-3-yl)methanol			X			
	Phenylacetic acid						X
Fatty acids and esters	Oleic acid			X			
Organic acids (non-sugar)	(3R,4S,5R)-Shikimic acid	X					
	Citric acid	X					X
	Glutaric acid						X
	Glyceric acid	X	X		X	X	X
	Malic acid	X	X	X	X	X	X
	Shikimic acid		X				
	Succinic acid					X	X
	Tartaric acid						X
	Tricarballylic acid		X	X			

Group	Compound	Atlas cedar cones	Mixed Conifer cones	Hazel-nut shells	Walnut shells	Coffee silver-skin	Cocoa shells
	Dehydroabietic acid	X					
	Hydroxydehydroabietic acid	X					
	1-Cyclohexene-1-carboxylic acid			X			
	Quinic acid	X			X		
Phenolics and polyphenols	Catechin						X
	Gallic acid			X	X		
	Protocatechuic acid		X	X			
	Vanillic acid		X	X	X		
Sugar acids	2,3,4-trihydroxybutyric acid				X		
	Arabonic acid					X	
	Galactaric acid					X	
	Galactonic acid					X	X
	Galacturonic acid					X	
	Glucaric acid					X	X
	Gluconic acid	X			X	X	X
	Mucic acid					X	
	Ribonic acid				X	X	X
Sugars and sugar alcohols	Arabinitol or Xylitol					X	
	Arabitol or Xylitol		X				
	D-Mannitol						X
	D-Pinitol	X	X				
	Erythritol						X
	Inositol	X	X	X		X	X
	Mannitol/Sorbitol	X					
	Myo-inositol	X					
	Ribitol or Arabitol						X
	Sucrose			X		X	X
	Sugars (unclassified)	X	X	X	X	X	X
	Sugar alcohol					X	X
	Xylitol or L-Arabitol						X

Table S3: Chemical compounds found in the ethanol extracts (Soxhlet) and organized by chemical group

Group	Compound	Atlas cedar cones	Mixed Conifer cones	Hazel-nut shells	Walnut shells	Coffee silver-skin	Cocoa shells
Amino acids and derivatives	L-5-Oxoprolin						X
Aromatic compounds	1-Phenanthrene carbaldehyde	X					
	Phenanthrene		X				
Fatty acids and esters	Arachidic acid					X	
	Behenic acid					X	
	Lignoceric acid					X	
	Linoleic acid	X	X			X	X
	Linolenic acid ethyl ester	X					
	Oleic acid	X	X	X		X	X
	Palmitic acid	X	X	X	X	X	X
	Palmitic acid methyl ester	X					
	Stearic acid	X	X	X		X	X
	Unknown fatty acid					X	X
Organic acids (non-sugar)	Tricarballylic acid						X
Phenolics and polyphenols	1,3,5-Trihydroxybenzene		X				
	Catechin		X				
	Protocatechuic acid		X				
	Trans-coniferyl alcohol			X			
	Vanillic acid					X	
	trans-Coniferyl alcohol				X		
Resin acids / diterpenoids	7-Oxo-dehydroabietic acid		X				
	7-Oxodehydroabietic acid					X	
	Abietic acid	X	X				
	Dehydroabietic acid	X	X			X	
	Isopimaric acid	X	X				
	Levopimaric acid	X					
	Neoabietic acid	X	X				
	Palustric acid	X	X				
	Pimaric acid	X	X				
Sterols	Beta-sitosterol					X	

Group	Compound	Atlas cedar cones	Mixed Conifer cones	Hazel-nut shells	Walnut shells	Coffee silver-skin	Cocoa shells
Sugar acids	Altronic acid						X
	Gluconic acid						X
	Gluconic or Manonic acid						X
Sugars and sugar alcohols	Arabinitol						X
	D-Mannitol						X
	Inositol						X
	Lactose						X
	Sugar		X				X

Table S4: Density of Organosolv extracts determined by Density Meter DE45 Delta Range™ (Mettler Toledo)

	Density (g/cm3)
Atlas cedar cones	0.9010
Conifer cones (mixed)	0.8983
Hazelnut shells	0.8971
Walnut shells	0.8983
Coffee silverskin	0.9020
Cocoa shells	0.9031

Table S5: Concentration in g/L of the Organosolv extracts obtained from agricultural and forest feedstocks

	Lignin (g/L)	Carbohydrates (g/L)	Others (g/L)	Total extractives (g/L)
Atlas cedar cones	8.584	1.773	4.913	15.27
Conifer cones (mixed)	10.76	2.114	1.589	14.47
Hazelnut shells	7.845	1.461	2.029	11.33
Walnut shells	8.956	1.574	4.095	14.62
Coffee silverskin	11.96	1.524	10.12	23.60
Cocoa shells	11.49	1.737	12.49	25.71

Table S6: Lignin composition (wt%) of the Organosolv extracts obtained from agricultural and forest feedstocks

	Lignin Composition (wt% of dry extract)		
	Acid insoluble lignin	Acid soluble lignin	Total Lignin
Atlas cedar cones	48.54	7.68	56.22
Conifer cones (mixed)	69.60	4.81	74.40
Hazelnut shells	66.37	2.84	69.21
Walnut shells	58.03	3.21	61.24
Coffee silverskin	36.38	14.30	50.68
Cocoa shells	26.27	18.40	44.67

Table S7: Carbohydrate composition (wt%) of the Organosolv extracts obtained from agricultural and forest feedstocks

	Carbohydrate Composition (wt% of dry extract)					
	Glucose	Xylose	Arabinose	Mannose	Galactose	Total
Atlas cedar cones	1.592	1.178	4.927	1.597	2.320	11.61
Conifer cones (mixed)	1.258	2.514	4.688	2.536	2.717	13.71
Hazelnut shells	0.514	8.469	1.477	0.264	2.165	12.89
Walnut shells	1.235	5.700	1.602	0.156	2.068	10.76
Coffee silverskin	1.302	0.421	2.380	0.313	2.042	6.46
Cocoa shells	2.543	0.118	1.611	0.448	2.035	6.75

a)

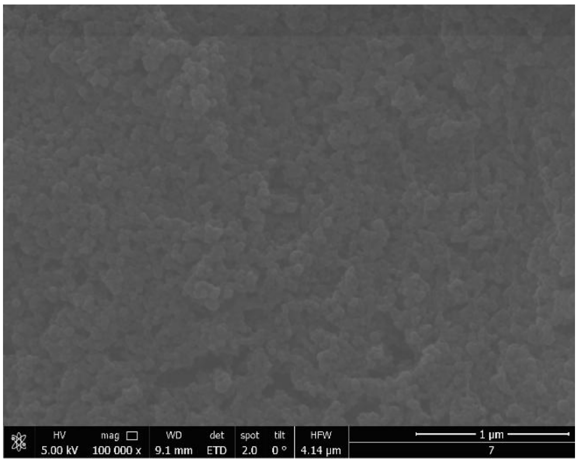

b)

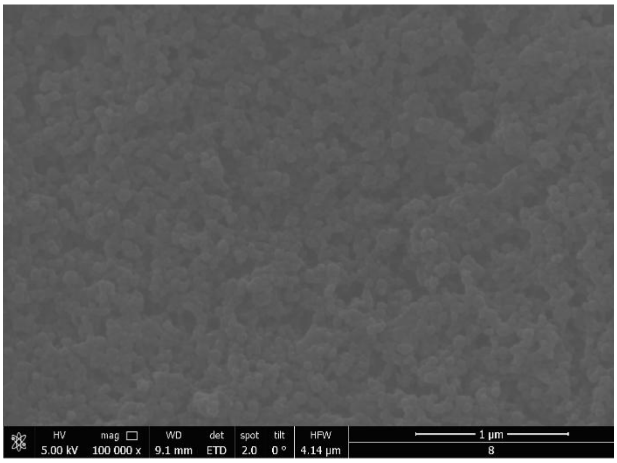

c)

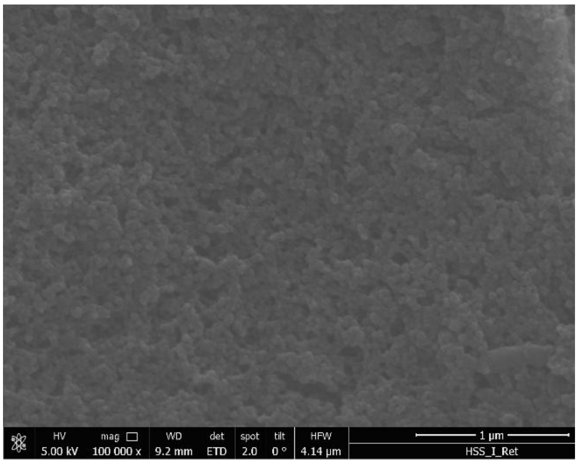

d)

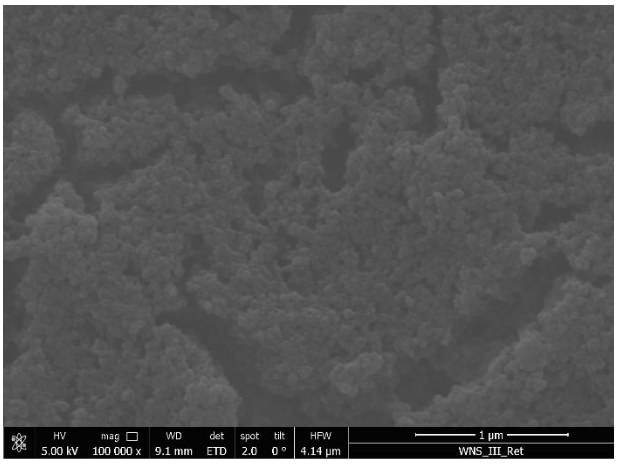

e)

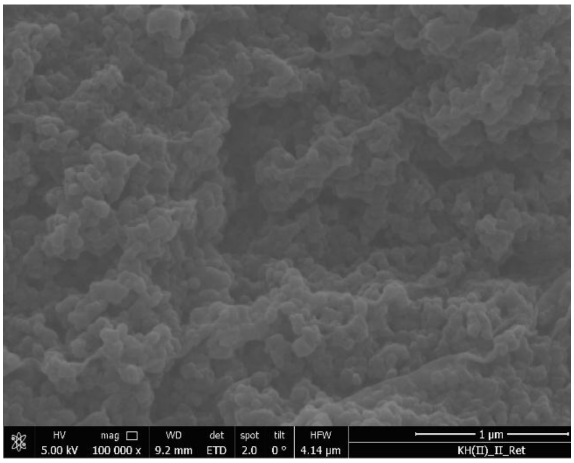

f)

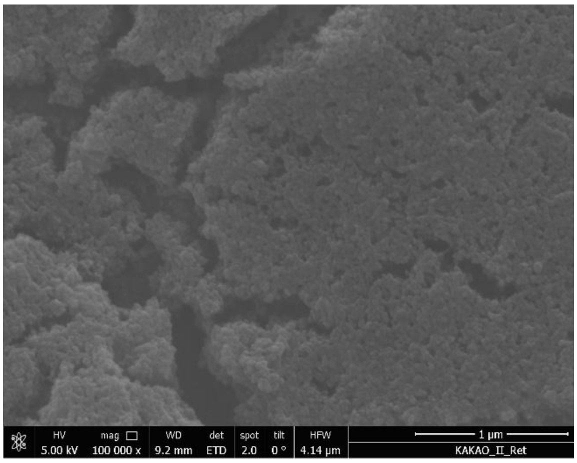

Figure S1: SEM pictures of a) Atlas cedar cones, b) Conifer cones (mixed), c) Hazelnut shells, d) Walnut shells, e) Coffee silverskins and f) Cocoa shells

Table S8: Conductivity Values of the permeates after ultrafiltration

	Conductivity Permeate 1 (µS/cm)	Conductivity Permeate 2 (µS/cm)
Atlas cedar cones	105.5 (± 2.3)	36.0 (± 9.7)
Conifer cones (mixed)	64.6 (± 2.2)	30.0 (± 1.6)
Hazelnut shells	85.8 (± 6.6)	32.9 (± 2.79
Walnut shells	121.4 (± 3.4)	50.2 (± 3.1)
Coffee silverskins	163.8 (± 5.3)	57.0 (± 7.5)
Cocoa shells	176.8 (± 5.2)	62.9 (± 3.1)

Table S9: Overall CLP production yield

	Yield extraction (wt%)	Yield precipitation and purification (wt%)	CLP yield (wt%)
Atlas cedar cones	16.86 ± 0.39	49.13 ± 2.55	8.285 ± 0.471
Conifer cones (mixed)*	16.07	47.78 ± 4.84	7.680 ± 0.778
Hazelnut shells	12.48 ± 0.10	43.33 ± 3.78	5.410 ± 0.474
Walnut shells	15.56 ± 0.21	47.51 ± 5.78	7.395 ± 0.890
Coffee silverskins	25.94 ± 0.21	31.28 ± 6.43	8.114 ± 1.670
Cocoa shells	28.90 ± 0.49	22.51 ± 4.84	6.503 ± 2.855

* only one extraction due to limited material available
